# Commercial determinants of health; accentuating positive, curtailing negative impacts: call for papers

**DOI:** 10.2471/BLT.26.295678

**Published:** 2026-02-01

**Authors:** Jintana Jankhotkaew, Monika Kosinska, Nisachol Cetthakrikul, Angkana Lekagul, Viroj Tangcharoensathien

**Affiliations:** a International Health Policy Program Foundation, Tiwanon Road, Nonthaburi, 11000, Thailand.; b Department of Health Determinants, Promotion and Prevention, World Health Organization, Geneva, Switzerland.

Commercial determinants of health are now recognized as a critical driver of health outcomes. These determinants encompass private sector activities and influences, which shape health across populations.^1^ For example, in 2019, approximately one third of global deaths were caused by tobacco, alcohol, ultra-processed foods and fossil fuels.^2^

The World Health Organization (WHO) defines commercial determinants of health as private sector activities that affect people’s health, directly or indirectly, positively or negatively.^3^ Expert consensus further proposes that commercial determinants of health include the systems, practices and pathways through which commercial actors drive social and health inequity.^2^ By interacting with social and political determinants of health, commercial determinants of health can reinforce inequities and contribute to poorer health outcomes.^2^

Profit-driven practices of health-harmful commercial actors, such as aggressive marketing, directly encourage excessive consumption, increase exposure to health risks and worsen health outcomes; they also indirectly influence policy in ways that weaken effective health protections.^2^ At the same time, commercial activity can also improve health. The development of affordable vaccines and medicines has saved millions of lives and helped people live longer.^3^ Societies can realize health gains by transforming commercial incentives and governance so that business models are aligned with, and accountable to, public health objectives.

A public health approach recognizes the need to address the harm created by commercial actors, and the need for robust systems approaches to support commercial practices that contribute to health improvements. Analyses of the commercial determinants of health can help reveal how power asymmetries, vested commercial interests and political economy dynamics influence both health outcomes and policy responses. While the adverse health impacts of major noncommunicable disease risk factors are well documented, the evidence base is much smaller for emerging commercially driven exposures, particularly petrochemicals, plastics and microplastics. Knowledge is also limited on how to harness the private sector’s potential to improve health while effectively managing conflicts of interest, ensuring accountability and protecting equity. 

The WHO Framework Convention on Tobacco Control (FCTC) remains the only legally binding instrument that directly addresses a major commercial health risk; however, how its legal and governance lessons can be adapted and transferred to other commercial determinants of health remains underexplored. Comparative lessons on how countries engage commercial actors to promote health, what works, under what conditions and with which safeguards, would be a valuable contribution to accelerating progress towards the sustainable development goals. 

The *Bulletin of the World Health Organization* calls for papers that examine the negative impacts of commercial determinants of health as well as those that explore how to incentivize and support the equitable distribution of positive contributions. The *Bulletin* welcomes papers that provide lessons learned from public–private collaborations in health. The *Bulletin* encourages submissions that go beyond the health risks of noncommunicable disease factors (such as tobacco, alcohol and health-harming diets) by focusing on other existing or emerging health risks, including petrochemicals, plastics and microplastics, social media, gambling, private equity, extractive industries and other commercial practices affecting health. For instance, microplastic exposure is suspected to adversely affect digestive, reproductive and respiratory systems;^4^ and exposure to pesticides increases the risk of Parkinson disease.^5^ Contributions may pay explicit attention to power, that is, how commercial actors shape markets, norms and policy processes in ways that influence health and health equity.^6^

The *Bulletin* welcomes papers on national legal instruments or regulations designed to govern the commercial determinants of health, including implementation lessons and good practices. Submissions may explore how provisions of the WHO FCTC and WHO-recommended cost-effective interventions for noncommunicable diseases^7^ contribute to developing instruments that address commercial determinants of health at the national level.

Commercial actors can contribute to health when their products align with health goals and where regulation exists, conflicts of interest are managed, and equity and access are prioritized. Papers examining the politics and political economy of green-energy restructuring and transitions towards climate neutrality, and their implications for public health, are particularly welcome.^8^ The *Bulletin* also encourages articles describing country experiences in promoting positive practices by commercial actors that contribute to health and health equity. Conditions required for success, such as regulation, tax and market incentives, secure procurement, integration into health promotion benefit packages and adequate funding support, may also be explored.

Manuscripts should be submitted in accordance with the *Bulletin’s* guidelines for contributors^9^ by 1 June 2026. This theme issue will be launched at the Prince Mahidol Award Conference in January 2027.
